# Amino Acid Frequency in the Proteome Is Not Associated With Realised Thermal Limit nor Dietary Niche Breadth in 35 Lepidoptera Species

**DOI:** 10.1002/ece3.72608

**Published:** 2025-12-03

**Authors:** Fernanda S. Caron, Zuzanna Pietras, Arkan Eddine‐Lomas, Rebecca von Hellfeld, Juliano Morimoto

**Affiliations:** ^1^ Programa de Pós‐graduação em Ecologia e Conservação Universidade Federal do Paraná Curitiba Brazil; ^2^ Department of Physics, Chemistry and Biology (IFM) Linköping University Linköping Sweden; ^3^ School of Biological Sciences University of Aberdeen Aberdeen UK; ^4^ Institute of Mathematics, School of Natural and Computing Sciences University of Aberdeen Aberdeen UK; ^5^ Department of Biological and Environmental Sciences, Ylistönrinne, Survontie 9 C (Ambiotica) University of Jyväskylä Jyväskylä Finland

**Keywords:** climate change, insect diversity, Lepidoptera, thermal ecology

## Abstract

Amino acids are the building blocks of proteins that perform essential physiological functions. Theory suggests that the proteome composition—the amino acid frequencies across all proteins in a genome—is associated with an organism's optimal growth temperature, offering insights into species' temperature limits. This hypothesis, however, is largely based on prokaryotic models and has not been thoroughly tested in complex multicellular eukaryotes, where many amino acids must be acquired through diet. Here, we integrated multiple databases and analysed amino acid frequencies in the proteomes of orthologous and non‐orthologous genes from 35 Lepidoptera species to test for correlations with maximum observed temperatures and diet breadth. Using a robust phylogenetic comparative approach, we found no evidence that proteome composition correlates with temperature or diet breadth, which are important ecological traits for Lepidopterans and affect their interactions with other species. These results suggest that, unlike in simpler organisms, animal proteome composition is shaped more strongly by intrinsic biophysical and energetic constraints than by ecological factors such as temperature exposure or dietary specialisation. Our study bridges evolutionary genetics with ecological physiology across a diverse group of insects and highlights the importance of publishing well‐designed null results. These findings also emphasise the limitations of using proteome composition as a proxy for ecological adaptation in multicellular species, while opening avenues of future research to further explore the complex interplay between genetics, physiology, and environment in shaping biodiversity.

## Graphical abstract

Amino acids form proteins that are crucial for life, and their distribution in an organism's proteome has been thought to reflect adaptations to temperature. By analysing proteome data from 35 Lepidoptera species, this study found no link between amino acid composition, temperature, or diet breadth. The results indicate that in complex animals, proteome composition is governed more by internal biophysical constraints than by ecological factors, highlighting the limits of using proteomic data to infer environmental adaptations.

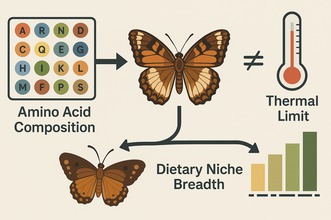

## 1 Introduction

The Anthropocene is marked by rising temperatures (Summerhayes and Zalasiewicz 2018), climate extremes (Pradhan et al. 2022), and biodiversity loss (Dirzo et al. 2014; Johnson et al. 2017; Turvey and Crees 2019). To survive, species must rapidly adapt to unpredictable conditions at the genomic, organismal, and behavioural levels, which is an unprecedented task in evolution (Bradshaw and Holzapfel 2006; Gienapp et al. 2008; Hoffmann and Sgrò 2011). Research has focused on understanding the correlations and causes associated with species' response to, and interaction with, their environment, seeking biological patterns that can be used to conserve what is left of our biodiversity (Mawdsley et al. 2009; Hannah 2010; Lancaster 2016).

Insects are particularly interesting subjects of insights in relation to thermal biology. Thermal adaptation in insects is correlated (although not necessarily causally) with changes in gene expression and ultimately, the genome. For instance, adaptation to warmer temperatures is known to trigger plastic transient increases in heat‐shock protein expression that, in the long term, lead to an increase in developmental rate and reduced genetic variability (Gilbert and Raworth 1996), all of which can contribute to changes in allele frequencies that modulate the genome composition (Quan et al. 2020; Mutamiswa et al. 2023). Importantly, insects tend to explore new environments to which they are not adapted in response to changes or fluctuations in thermal environments (Franks and Hoffmann 2012). This leads to rapid evolutionary change that facilitates thermal adaptation but that can also shape the genome through adaptations to this novel environment, particularly in relation to diet which is a major ecological factor for insects (Gilchrist and Lee 2007; Mutamiswa et al. 2023). Indeed, locally adapted *Drosophila melanogaster* populations in a latitudinal cline with varying thermal exposure display population‐specific dietary responses (Zanco et al. 2025). However, there are still many aspects of organismal biology and its relationship to the environment that remain unexplored (Merilä 2012; Bozinovic and Pörtner 2015).

One such case is the environmental effects on the proteome—that is, the collection and frequency of amino acids from coding sequences of a genome, also referred to as ‘exome’ (Du et al. 2018). Theory predicts that changes in amino acid frequencies across proteomes are associated with optimal growth temperatures of the species, and past empirical work has shown that mesophilic and thermophilic prokaryotes indeed have distinct amino acid frequencies in their proteomes (Dufton 1997; Seligmann 2003; Singer and Hickey 2003; Tekaia and Yeramian 2006; Swire 2007). Work from our group has shown that amino acid frequencies in the proteomes of three domains of life (archaea, bacteria, and eukaryotes) respond to growth temperature, although this effect appears to be relatively small (Morimoto and Pietras 2024). Nevertheless, proteome composition can be linked to life‐history traits under selection, such as lifespan (Morimoto and Pietras 2025). Thus, these findings suggest that proteome composition is sensitive to temperature, is correlated to life‐history traits under selection and therefore, can have signs of adaptation to ecological conditions. To date, however, we lack studies to investigate the effects of temperature on proteome composition in multicellular eukaryotes (Roberts 1999; but see e.g., Jensen et al. 2021). This is because in multicellular organisms—especially ectotherms—often there are microclimatic conditions that can dilute our ability to ascertain with precision optimal growth temperatures (Pincebourde et al. 2007; Kemppinen et al. 2024). As a result, even though there are efforts to create databases for optimal growth temperature for prokaryotes (e.g., Helena‐Bueno et al. 2021), we lack similar resources for multicellular eukaryotes. An alternative is to use macroclimatic information as proxies for species' thermal tolerance and limits since, at least in insects, they interact and are both important to determine their macroecological distribution and occupancy (König et al. 2024). However, to our knowledge, there have been no studies investigating the thermal biology with proteomes in multicellular eukaryotes.

In addition to temperature, diet also plays a role in thermal adaptation (Hardison and Eliason 2024) and as a result, the proteome can also evolve and be shaped in response to diet (Luca et al. 2010; Birnbaum and Abbot 2020). Recent studies which focused on the proteome of multicellular eukaryotes have shown that the amino acid frequencies in fruit flies ( *Drosophila melanogaster* ) and mice ( *Mus musculus* ) contain information about their dietary requirements (Piper et al. 2017; Gómez Ortega et al. 2023). Matching the dietary amino acid availability to the proteome amino acid frequencies improved reproduction without associated lifespan costs to flies and improved the growth rate in mice (Piper et al. 2017). This effect in flies was observed so long as the amino acid availability in the diet matched the absolute amino acid frequencies of the proteome, not the amino acid frequencies weighted by gene transcription (Gómez Ortega et al. 2023). Feeding is one process among many through which organisms interact with their environments, and diet availability and quality are known to be affected by climate change (Stephens and Krebs 1986; Simpson and Raubenheimer 2012; Rosenblatt and Schmitz 2016; Macdiarmid and Whybrow 2019; European Food Safety Authority (EFSA) et al. 2020). Feeding allows multicellular organisms to acquire essential amino acids that compose proteins and ultimately, the proteome (Simpson and Raubenheimer 2012; Piper et al. 2017). In insect pollinators, it is known that temperature modulates the availability of nutrients (Shrestha et al. 2018). Therefore, it is plausible, although untested, that proteomes with certain compositions belong to generalist (or specialist) species which can tolerate higher temperatures. This would result in an association between proteome composition, diet breadth, and the maximum temperature in which species are encountered. Finding such patterns would be groundbreaking since proteome data are relatively cheap to obtain and there are well‐curated databases available for investigations, as opposed to time‐consuming, labour‐intensive experiments to ascertain diet breadth and thermal limits.

**FIGURE 1:**
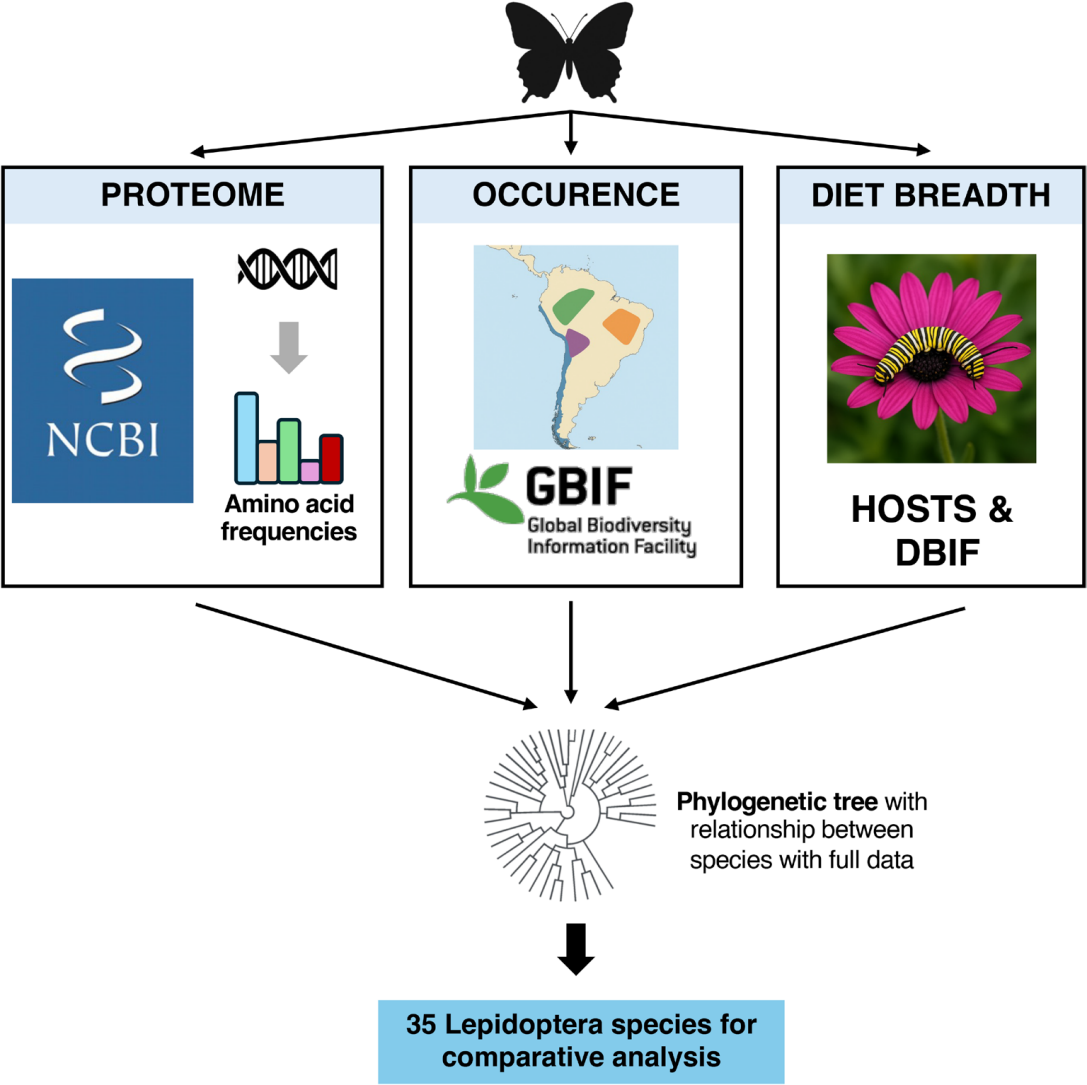
Data integration for hypothesis testing. We integrated data from several databases to gain a full understanding of how amino acid frequency in the proteome correlates with diet breadth and realised thermal niche from occurrence data. Data for the proteome, occurrence and diet breadth of Lepidoptera species were obtained from NCBI, GBIF, HOSTS, and DBIF. This resulted in the final number of 35 species for which we had a complete dataset (i.e., information on amino acid frequencies *and* occurrences *and* diet breadth).

In this study, we set out to test whether two key ecological traits—realised thermal limit and dietary breadth—are correlated with the proteome composition of 35 Lepidoptera species. Herbivorous insects such as Lepidopterans are ideal models to test the effects of temperature and diet on proteomes for three reasons. First, Lepidopterans and other arthropods are particularly vulnerable to climate change (Harvey et al. 2023), have been declining at an incredible pace (Habel et al. 2019; Warren et al. 2021), but are often overlooked in conservation policies (Duffus and Morimoto 2022). Second, the effects of climate change and urbanisation on biodiversity loss appear to disproportionately affect diet specialist species, which may be more vulnerable to rapid and unpredictable changes in food supply or quality (Merckx and Van Dyck 2019; Hulshof et al. 2024). And third, there are multiple fully annotated genomes available for butterflies and other insects, making the study of amino acid profiles across proteomes feasible at larger scales (Espeland et al. 2018; Liu et al. 2020; Kawahara et al. 2023). Thus, better understanding how Lepidopteran proteomes correlate (or not) with ecological traits can open fruitful avenues of fundamental and applied research on insect physiology, ecology, and conservation. The 35 species of Lepidopterans were here selected due to the availability of data across different databases, which enabled data integration about their amino acid frequencies in the proteome (NCBI database), their dietary breadth (HOSTS database) and their occurrence (GBIF database) (Figure 1). We acknowledge this is only a subset of all Lepidopterans for which data are available within each individual database, but we opted to conduct an analysis of species for which full data integration was possible as opposed to using alternative methods such as imputation. This consolidated data allowed us to test whether amino acid frequencies in the proteome were associated with species' diet breadth, the recorded maximum temperature where each species was observed (henceforth ‘climatic high temperatures’), or an interaction between them.

As there has been no prior study linking proteome with temperature or diet breadth, we based our hypothesis on the prokaryote literature. Specifically, we expected thermolabile amino acids such as cysteine to be negatively correlated with maximum temperature (Seligmann 2003; Singer and Hickey 2003; Hickey and Singer 2004; Tekaia and Yeramian 2006; Swire 2007; Morimoto and Pietras 2024). We also hypothesised—albeit without literature to support this—that narrower dietary breadth could be positively and negatively associated with at least some amino acids (although there is no reason to a priori pinpoint the specific amino acids), because we reasoned that diet specialists would have proteomes with highly specialised compositions as opposed to the more balanced proteome of generalists. This could drive the relationship between certain amino acids positively or negatively. To test our hypotheses, we first analysed whether the overall amino acid frequencies in the proteomes correlated with the maximum temperature at which species occur and species' diet breadth. Next, we separated the amino acid frequencies of orthologous and non‐orthologous genes. Our rationale was that, due to differences in evolutionary patterns and selective pressures, these two classes of genes could give us different insights into the relationship between proteomes and species' ecology. This is because we know that orthologs and non‐orthologs can be under different selective pressures (e.g., Przytycka et al. 2008). As a result, we predicted that because orthologous genes are often under selection to maintain ancestral functions across multiple species (Gabaldón and Koonin 2013), they could be less likely to diversify to the extent that allows us to capture associations with species' temperature limits or diet breadth. Conversely, non‐orthologous genes could evolve and gain functions in specific lineages, providing a better subset of the proteome to identify correlations with species' temperature limits or diet breadth. Our work provides a unique and innovative perspective onto evolutionary genomics, opening future avenues of research into the links between proteome composition and life‐history and ecological traits.

## 2 Materials and Methods

### 2.1 Proteome Analysis

The NCBI database from which the proteome for the 35 Lepidoptera species was retrieved was accessed on or before 6 January 2024 (Morimoto and Pietras 2024). We retrieved proteome information for species which had complete and annotated reference sequence (RefSeq) genomes with suitable taxonomic identification in the NCBI database. This approach ensured that our estimates of amino acid frequencies were true representatives of amino acids from coding sequences. The proteome fasta files were downloaded from FTP servers and were processed in the statistical software R version 4.3.2 (R Core Team 2013) to estimate amino acid profiles. Because of the positive correlation between the number of redundant codons and the frequency of amino (Estimate: 0.0102, SE: 0.00032; *t*‐value: 32.08; *p* < 0.001), we standardised our amino acid profiles, dividing amino acid frequency by the number of redundant codons. For all analyses, we normalised amino acid frequencies using the ‘scale()’ function in R with default parameters. This, and the following steps of the methodology, are exemplified in Figure 1.

### 2.2 Orthologous Analysis

Inferring orthologous and non‐orthologous genes from the proteomes of the studied species requires effective algorithms which form accurate gene trees. OrthoFinder is a recently developed orthologue inference algorithm that has innovated in providing more accurate ortholog inferences without sacrificing time costs (Emms and Kelly 2019). OrthoFinder's algorithm was run on the collective Lepidoptera proteomes and inferred orthologs from orthogroup trees it produced. Within OrthoFinder's default algorithm, the sequence search method used was DIAMOND (Buchfink et al. 2015) and the orthogroup tree inference method used was DendroBlast (Kelly and Maini 2013). This method has been proven to achieve accurate results with low run time costs (Emms and Kelly 2015; Altenhoff et al. 2016). Amino acid frequency for each orthologous and non‐orthologous gene in each species as well as for each species' whole proteome was calculated for the phylogenetic comparative analysis.

### 2.3 Phylogenetic Reconstruction

**FIGURE 2:**
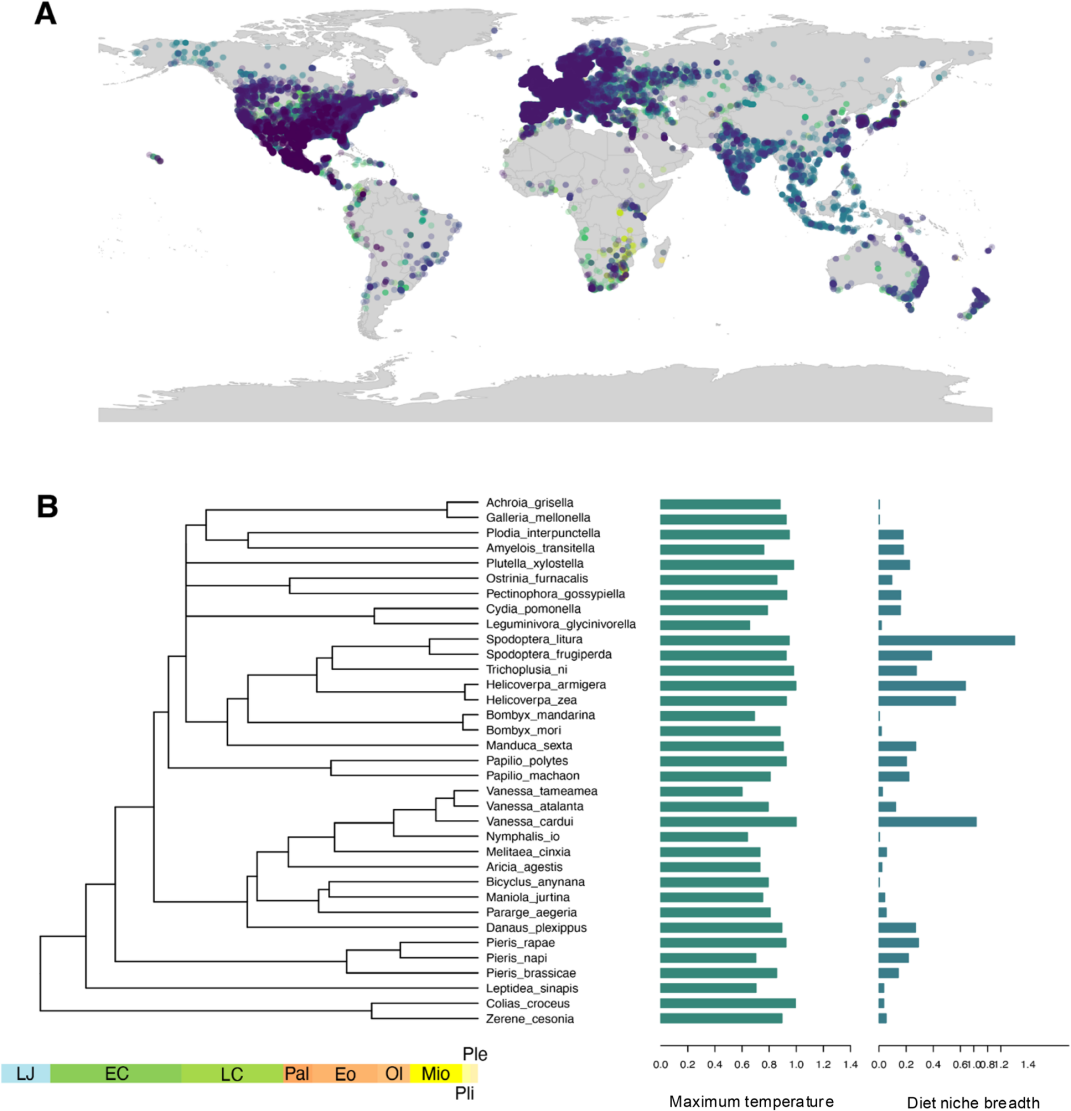
Ecological traits and phylogenetic relationships. (A) Coverage of our observation points for the 35 species from which maximum temperature and diet breadth were used to test associations with proteome composition. (B) Phylogenetic reconstruction of the relationship between the 35 species in our database (including 2 outgroups) along with the distribution of maximum temperature and dietary niche breadth of each species (right‐hand side bars).

To assess the correlation between Lepidoptera's traits, we needed to reconstruct the phylogeny of the species in the present study. To do this, we obtained COI sequences from each species from GenBank (last accessed 20 August 2024; Table S1). Additionally, we obtained sequences for two outgroup species ( *Cheumatopsyche brevilineata* and *Hydromanicus wulaianus*) that allowed us to root the estimated phylogeny. The sequences were aligned using an auto strategy in MAFFT online service v.7 (Kuraku et al. 2013; Katoh et al. 2019). Then, we reconstructed the phylogenetic relationships of the species on IQ‐TREE v.2.3.6 (Minh et al. 2020). Simultaneously, we ran ModelFinder using the option ‐m MFP + MERGE to find the best evolutionary model for each codon position (Kalyaanamoorthy et al. 2017). We also ran an ultrafast bootstrap (Hoang et al. 2018) with 1000 replicates with branch lengths to assess phylogenetic uncertainty in the subsequent analyses. The resulting maximum likelihood tree did not show unusually long branches that could indicate poor alignment or obvious identification errors in some species. Finally, all bootstrap replicates were calibrated with PATHd8 (Britton et al. 2007). The calibrations used were obtained from Kawahara et al. (2023). As these authors used several distinct calibration schemes, we chose the calibration scheme that they used in their subsequent analyses while filtering (subsetting) the nodes we had present in our phylogeny (Table S2; Figure 2). We assigned a maximum age and a minimum age to each calibration, except for the oldest calibration. This is due to the requirement of PATHd8 that at least one calibration be fixed.

### 2.4 Ecological and Occurrence Data

We retrieved and consolidated diet breadth data from two databases on 6 March 2024: (1) HOSTS, a database on host plants for insects by the Natural History Museum in London (Robinson et al. 2010) and (2) DBIF, also a resource for recorded host plants for insects which have been recorded in Great Britain (Smith and Roy 2008; Ward et al. 2019). There are limitations to using these public databases, such as the potential for taxonomic misidentification of either plants or insects, but they provide an invaluable resource to explore biological phenomena at larger scales (see e.g., Padovani et al. 2020). We estimated diet breadth at the genus level; that is, the number of different plant genera that a butterfly species has been recorded using as a food source. Occurrences of each species were retrieved from GBIF (https://doi.org/10.15468/dl.q74xtz, 11 March 2024; Figure 1). Climatic variables where each species occurs were obtained using the worldclim_global function with a resolution of 2.5 in the *geodata* package v.0.6‐2 (Hijmans et al. 2023). For the subsequent analyses, we calculated the maximum temperature that each species has been found as a proxy of their thermal limit using the bio5 variable (maximum temperature of warmest month). Our rationale was that the maximum upper temperature where the species has been observed represented an upper limit on their thermal tolerance.

### 2.5 Phylogenetic Comparative Analyses

We performed a Phylogenetic Least Squares Regression (PGLS) to assess the effect of diet and temperature on amino acid counts. Diet breadth and maximum temperature were log‐transformed prior to the analyses. A PGLS analysis was done for the thermal limit and diet breadth, repeated for the 1000 topology replicates to account for phylogenetic uncertainty. We ran a PGLS for each amino acid separately as their frequencies vary widely. We repeated the PGLS three times considering the amino acid frequency of all genes pooled together at first, then only orthologous or non‐orthologous genes. To control for multiple comparisons, we adjusted the *p*‐value reported in this study by using the ‘p.adjust()’ function specifying the Benjamini and Hochberg (1995) correction method.

It is important to mention that this study integrates species' data from multiple databases (i.e., NCBI, GBIF, HOSTS). The absence of data in any of these databases precludes us from using the species in the analysis. For example, if we lacked data on species' diet breadth, we had to exclude this species from the final analysis even if they had a RefSeq genome available to estimate amino acid frequencies in their proteome. Thus, even though there are many RefSeq genomes from butterflies, including their well‐established phylogenetic relationships (e.g., Cicconardi et al. 2023), most of those species lack data on their diet breadth and/or distribution. Frankly, diet breadth data is often the most constraining data to obtain for our analysis. Thus, our final sample size of 35 cosmopolitan Lepidoptera species is a result of this trade‐off between statistical power and the ability to integrate multiple databases with complete information. The continuing data collection and recording of butterflies will help improve this resolution.

## 3 Results

Our PGLS showed no evidence that individual amino acid frequencies in the proteome correlated with maximum temperature or diet breadth (Table 1). This was consistent for the analysis of the entire proteome (all genes), orthologous, and non‐orthologous genes (Table 1). Together, these results show that proteome composition is not correlated with ecological traits related to thermal limit and diet breadth in the 35 species of cosmopolitan Lepidopterans analysed in this study.

**TABLE 1:** No evidence that proteome composition is associated with ecological traits in 35 butterfly species.

Amino acid	Intercept	Diet niche breadth				Maximum temperature				*R* ^2^
		Estimate	Standard error	*t*‐value	*p*	Estimate	Standard error	*t*‐value	*p* *	
All genes										
M	8.445 (7.14 to 9.82)	0.179 (0.134 to 0.217)	0.118 (0.11 to 0.124)	1.517 (1.14 to 1.87)	1 (1–1)	−2.403 (−2.81 to −2.025)	1.057 (0.996 to 1.136)	−2.281 (−2.619 to −1.866)	1 (0.802–1)	0.144 (0.101 to 0.193)
L	3.038 (2.007 to 3.942)	−0.101 (−0.127 to −0.056)	0.094 (0.088 to −0.099)	−1.067 (−1.352 to 0.597)	1 (1–1)	−0.705 (−1.026 to −0.403)	0.846 (0.8 to 0.917)	−0.836 (−1.23 to −0.463)	1 (1–1)	0.191 (0.126 to 0.237)
V	0.267 (−1.013 to 2.451)	0.029 (−0.012 to 0.067)	0.112 (0.105 to 0.12)	0.26 (−0.098 to 0.622)	1 (1–1)	−0.133 (−0.753 to 0.212)	1.012 (0.946 to 1.091)	−0.132 (−0.703 to 0.218)	1 (1–1)	0.005 (0 to 0.022)
A	−2.765 (−3.439 to −2.064)	−0.005 (−0.038 to 0.013)	0.102 (0.096 to 0.108)	−0.051 (−0.376 to 0.128)	1 (1–1)	0.685 (0.462 to 0.979)	0.916 (0.862 to 0.997)	0.747 (0.488 to 1.094)	1 (1–1)	0.038 (0.016 to 0.057)
R	−4.758 (−6.603 to −3.576)	0.042 (0.006 to 0.082)	0.144 (0.135 to 0.153)	0.293 (0.043 to 0.556)	1 (1–1)	1.228 (0.895 to 1.716)	1.291 (1.208 to 1.408)	0.944 (0.703 to 1.262)	1 (1–1)	0.093 (0.051 to 0.175)
F	8.183 (7.263 to 9.247)	0.125 (0.103 to 0.152)	0.121 (0.113 to 0.127)	1.033 (0.858 to 1.269)	1 (1–1)	−2.288 (−2.587 to −2.024)	1.084 (1.028 to 1.16)	−2.115 (−2.431 to −1.772)	1 (1–1)	0.141 (0.093 to 0.187)
Y	4.752 (3.632 to 5.809)	−0.087 (−0.117 to −0.064)	0.123 (0.115 to 0.13)	−0.713 (−0.953 to −0.513)	1 (1–1)	−1.155 (−1.451 to −0.833)	1.107 (1.036 to 1.192)	−1.049 (−1.318 to −0.72)	1 (1–1)	0.169 (0.104 to 0.247)
P	−2.605 (−3.475 to −1.837)	−0.016 (−0.05 to 0.01)	0.111 (0.102 to 0.119)	−0.149 (−0.441 to 0.097)	1 (1–1)	0.657 (0.411 to 0.95)	0.993 (0.925 to 1.087)	0.664 (0.396 to 0.975)	1 (1–1)	0.023 (0.01 to 0.042)
I	1.066 (0.311 to 1.696)	−0.028 (−0.053 to −0.001)	0.101 (0.095 to 0.107)	−0.277 (−0.531 to −0.013)	1 (1–1)	−0.222 (−0.443 to 0.015)	0.905 (0.844 to 0.99)	−0.245 (−0.498 to 0.015)	1 (1–1)	0.018 (0.007 to 0.028)
S	1.281 (0.338 to 2.54)	−0.033 (−0.076 to 0.01)	0.114 (0.106 to 0.121)	−0.29 (−0.646 to 0.094)	1 (1–1)	−0.257 (−0.716 to 0.056)	1.02 (0.958 to 1.119)	−0.252 (−0.676 to 0.051)	1 (1–1)	0.021 (0.012 to 0.034)
T	−4.117 (−5.202 to −2.635)	−0.063 (−0.092 to −0.025)	0.119 (0.11 to 0.129)	−0.524 (−0.782 to −0.216)	1 (1–1)	1.103 (0.751 to 1.39)	1.074 (0.989 to 1.196)	1.025 (0.702 to 1.298)	1 (1–1)	0.036 (0.018 to 0.061)
Q	1.932 (0.315 to 3.226)	0.007 (−0.049 to 0.054)	0.137 (0.126 to 0.149)	0.051 (−0.357 to 0.406)	1 (1–1)	−0.52 (−0.898 to −0.087)	1.235 (1.126 to 1.379)	−0.421 (−0.74 to −0.065)	1 (1–1)	0.013 (0.003 to 0.025)
G	−3.593 (−5.319 to −2.17)	0.065 (0.034 to 0.096)	0.129 (0.119 to 0.135)	0.502 (0.266 to 0.74)	1 (1–1)	0.889 (0.544 to 1.348)	1.158 (1.102 to 1.22)	0.768 (0.467 to 1.18)	1 (1–1)	0.096 (0.064 to 0.15)
E	4.044 (2.577 to 5.785)	0.156 (0.119 to 0.189)	0.121 (0.113 to 0.128)	1.285 (0.963 to 1.591)	1 (1–1)	−1.203 (−1.675 to −0.828)	1.088 (1.031 to 1.171)	−1.108 (−1.487 to −0.76)	1 (1–1)	0.051 (0.03 to 0.078)
D	2.793 (1.042 to 4.327)	−0.009 (−0.06 to 0.033)	0.136 (0.128 to 0.146)	−0.069 (−0.441 to 0.247)	1 (1–1)	−0.72 (−1.142 to −0.297)	1.23 (1.146 to 1.342)	−0.586 (−0.886 to −0.245)	1 (1–1)	0.029 (0.011 to 0.071)
H	0.319 (−0.45 to 1.066)	−0.138 (−0.175 to −0.104)	0.097 (0.092 to 0.103)	−1.413 (−1.791 to −1.078)	1 (1–1)	0.046 (−0.216 to 0.293)	0.876 (0.814 to 0.962)	0.053 (−0.237 to 0.339)	1 (1–1)	0.124 (0.086 to 0.166)
K	1.028 (0.222 to 1.805)	0.146 (0.104 to 0.179)	0.131 (0.124 to 0.138)	1.11 (0.813 to 1.376)	1 (1–1)	−0.405 (−0.624 to −0.154)	1.176 (1.106 to 1.294)	−0.342 (−0.541 to −0.13)	1 (1–1)	0.055 (0.031 to 0.075)
W	−1.186 (−4.242 to 0.768)	0.169 (0.124 to 0.223)	0.161 (0.145 to 0.176)	1.046 (0.814 to 1.299)	1 (1–1)	0.22 (−0.316 to 1.041)	1.451 (1.32 to 1.608)	0.153 (−0.227 to 0.695)	1 (1–1)	0.092 (0.057 to 0.166)
C	−0.098 (−1.063 to 1.08)	−0.223 (−0.257 to −0.19)	0.118 (0.108 to 0.129)	−1.896 (−2.076 to −1.663)	1 (1–1)	0.273 (−0.077 to 0.569)	1.065 (0.971 to 1.181)	0.261 (−0.068 to 0.524)	1 (1–1)	0.177 (0.151 to 0.209)
N	0.397 (−0.326 to 0.986)	−0.058 (−0.079 to −0.038)	0.097 (0.091 to 0.103)	−0.594 (−0.814 to 0.381)	1 (1–1)	−0.011 (−0.208 to 0.199)	0.874 (0.819 to 0.953)	−0.013 (−0.243 to 0.22)	1 (1–1)	0.027 (0.018 to 0.036)
Ortholog genes										
M	7.941 (6.55 to 9.211)	0.172 (0.129 to 0.208)	0.115 (0.107 to 0.12)	1.498 (1.118 to 1.851)	1 (1–1)	−2.256 (−2.625 to −1.848)	1.028 (0.972 to 1.103)	−2.195 (−2.544 to −1.755)	1 (0.959–1)	0.135 (0.09 to 0.179)
L	2.532 (1.291 to 3.507)	−0.091 (−0.118 to −0.045)	0.096 (0.09 to 0.101)	−0.944 (−1.225 to −0.473)	1 (1–1)	−0.564 (−0.91 to −0.212)	0.865 (0.817 to 0.936)	−0.653 (−1.084 to −0.235)	1 (1–1)	0.144 (0.09 to 0.18)
V	−0.268 (−1.372 to 1.478)	0.027 (−0.011 to 0.061)	0.104 (0.098 to 0.111)	0.262 (−0.107 to 0.618)	1 (1–1)	0.021 (−0.479 to 0.311)	0.939 (0.881 to 1.013)	0.022 (−0.489 to 0.345)	1 (1–1)	0.007 (0 to 0.027)
A	−2.812 (−3.474 to −2.102)	−0.01 (−0.043 to 0.008)	0.102 (0.096 to 0.108)	−0.096 (−0.423 to 0.084)	1 (1–1)	0.703 (0.477 to 0.998)	0.916 (0.863 to 0.998)	0.766 (0.503 to 1.111)	1 (1–1)	0.036 (0.015 to 0.056)
R	−4.367 (−5.888 to −3.321)	0.045 (0.013 to 0.079)	0.135 (0.127 to 0.143)	0.335 (0.098 to 0.575)	1 (1–1)	1.113 (0.828 to 1.525)	1.214 (1.144 to 1.321)	0.913 (0.683 to 1.209)	1 (1–1)	0.094 (0.052 to 0.168)
F	7.868 (6.895 to 8.921)	0.125 (0.103 to 0.153)	0.121 (0.113 to 0.127)	1.034 (0.862 to 1.268)	1 (1–1)	−2.198 (−2.496 to −1.916)	1.086 (1.031 to 1.162)	−2.026 (−2.358 to −1.661)	1 (1–1)	0.129 (0.083 to 0.171)
Y	4.21 (3.028 to 5.249)	−0.093 (−0.121 to −0.07)	0.123 (0.115 to 0.13)	−0.757 (−0.994 to −0.555)	1 (1–1)	−1.005 (−1.295 to −0.67)	1.106 (1.037 to 1.19)	−0.91 (−1.18 to −0.575)	1 (1–1)	0.154 (0.093 to 0.225)
P	−2.412 (−3.299 to −1.655)	−0.006 (−0.037 to 0.019)	0.104 (0.097 to 0.111)	−0.054 (−0.352 to 0.183)	1 (1–1)	0.592 (0.352 to 0.891)	0.938 (0.88 to 1.019)	0.633 (0.375 to 0.957)	1 (1–1)	0.026 (0.012 to 0.045)
I	1.004 (0.224 to 1.673)	−0.025 (−0.05 to 0.002)	0.099 (0.093 to 0.105)	−0.25 (−0.506 to 0.021)	1 (1–1)	−0.206 (−0.429 to 0.04)	0.893 (0.833 to 0.976)	−0.231 (−0.494 to 0.042)	1 (1–1)	0.016 (0.005 to 0.026)
S	1.701 (0.875 to 2.785)	0.006 (−0.033 to 0.05)	0.105 (0.098 to 0.111)	0.055 (−0.308 to 0.467)	1 (1–1)	−0.411 (−0.854 to −0.134)	0.937 (0.882 to 1.025)	−0.435 (−0.883 to −0.137)	1 (1–1)	0.013 (0.006 to 0.028)
T	−4.689 (−5.845 to −3.114)	−0.056 (−0.086 to −0.015)	0.116 (0.109 to 0.125)	−0.471 (−0.754 to −0.131)	1 (1–1)	1.246 (0.878 to 1.554)	1.048 (0.975 to 1.155)	1.184 (0.851 to 1.479)	1 (1–1)	0.054 (0.03 to 0.089)
Q	2.466 (0.893 to 3.808)	−0.013 (−0.069 to 0.032)	0.135 (0.124 to 0.147)	−0.098 (−0.511 to 0.253)	1 (1–1)	−0.644 (−1.041 to −0.216)	1.216 (1.11 to 1.354)	−0.533 (−0.861 to −0.179)	1 (1–1)	0.03 (0.009 to 0.051)
G	−3.565 (−5.239 to −2.266)	0.034 (0.006 to 0.064)	0.126 (0.115 to 0.131)	0.271 (0.047 to 0.514)	1 (1–1)	0.896 (0.552 to 1.348)	1.125 (1.073 to 1.183)	0.795 (0.475 to 1.217)	1 (1–1)	0.073 (0.048 to 0.118)
E	4.498 (2.992 to 6.463)	0.145 (0.108 to 0.18)	0.122 (0.113 to 0.13)	1.193 (0.866 to 1.518)	1 (1–1)	−1.309 (−1.839 to −0.927)	1.096 (1.035 to 1.176)	−1.196 (−1.619 to −0.851)	1 (1–1)	0.05 (0.029 to 0.08)
D	2.544 (0.831 to 3.836)	−0.021 (−0.071 to 0.023)	0.131 (0.123 to 0.14)	−0.162 (−0.543 to 0.177)	1 (1–1)	−0.634 (−0.989 to −0.236)	1.182 (1.099 to 1.291)	−0.532 (−0.84 to −0.205)	1 (1–1)	0.032 (0.012 to 0.07)
H	0.341 (−0.406 to 1.035)	−0.123 (−0.155 to −0.091)	0.093 (0.088 to 0.099)	−1.31 (−1.676 to −0.985)	1 (1–1)	0.033 (−0.217 to 0.262)	0.838 (0.781 to 0.922)	0.039 (−0.249 to 0.313)	1 (1–1)	0.11 (0.073 to 0.15)
K	1.072 (0.291 to 1.835)	0.14 (0.1 to 0.173)	0.129 (0.122 to 0.136)	1.079 (0.789 to 1.346)	1 (1–1)	−0.403 (−0.626 to −0.161)	1.158 (1.092 to 1.271)	−0.345 (−0.551 to −0.137)	1 (1–1)	0.052 (0.028 to 0.071)
W	−0.119 (−2.559 to 1.584)	0.084 (0.034 to 0.138)	0.151 (0.141 to 0.162)	0.557 (0.217 to 0.925)	1 (1–1)	0.001 (−0.482 to 0.67)	1.361 (1.272 to 1.479)	0.001 (−0.364 to 0.477)	1 (1–1)	0.023 (0.005 to 0.074)
C	0.216 (−0.655 to 1.339)	−0.2 (−0.229 to −0.167)	0.108 (0.099 to 0.116)	−1.865 (−2.074 to −1.583)	1 (1–1)	0.171 (−0.167 to 0.434)	0.97 (0.895 to 1.065)	0.177 (−0.171 to 0.44)	1 (1–1)	0.181 (0.154 to 0.213)
N	0.444 (−0.291 to 1.031)	−0.055 (−0.075 to −0.034)	0.098 (0.092 to 0.104)	−0.553 (−0.773 to −0.344)	1 (1–1)	−0.031 (−0.226 to 0.179)	0.88 (0.824 to 0.961)	−0.035 (−0.258 to 0.199)	1 (1–1)	0.025 (0.016 to 0.034)
Non‐ortholog genes										
M	12.406 (6.236 to 18.547)	0.005 (−0.078 to 0.141)	0.24 (0.194 to 0.265)	0.021 (−0.312 to 0.72)	1 (1–1)	−3.385 (−5.146 to −1.748)	2.142 (1.937 to 2.296)	−1.568 (−2.628 to −0.82)	1 (0.785–1)	0.159 (0.044 to 0.278)
L	9.584 (5.364 to 13.66)	−0.176 (−0.297 to −0.063)	0.27 (0.249 to 0.293)	−0.653 (−1.052 to −0.243)	1 (1–1)	−2.453 (−3.621 to −1.262)	2.428 (2.264 to 2.664)	−1.009 (−1.492 to −0.503)	1 (1–1)	0.156 (0.057 to 0.258)
V	−5.623 (−11.058 to 2.09)	−0.198 (−0.28 to −0.089)	0.223 (0.178 to 0.248)	−0.889 (−1.164 to −0.498)	1 (1–1)	1.678 (−0.505 to 3.145)	1.989 (1.775 to 2.149)	0.843 (−0.282 to 1.513)	1 (1–1)	0.032 (0.013 to 0.08)
A	−4.822 (−7.315 to −0.747)	0.234 (0.173 to 0.283)	0.177 (0.151 to 0.191)	1.328 (1.087 to 1.577)	1 (1–1)	1.078 (−0.023 to 1.789)	1.576 (1.466 to 1.66)	0.681 (−0.016 to 1.131)	1 (1–1)	0.211 (0.129 to 0.293)
R	−4.594 (−12.058 to −0.732)	0.206 (0.124 to 0.318)	0.245 (0.215 to 0.286)	0.841 (0.558 to 1.161)	1 (1–1)	0.999 (−0.023 to 3.013)	2.215 (1.973 to 2.54)	0.451 (−0.012 to 1.275)	1 (1–1)	0.101 (0.037 to 0.233)
F	21.72 (18.539 to 24.326)	0.076 (−0.003 to 0.157)	0.194 (0.18 to 0.205)	0.394 (−0.017 to 0.842)	1 (1–1)	−5.955 (−6.719 to −5.122)	1.739 (1.645 to 1.858)	−3.442 (−3.886 to −2.823)	0.098 (0.029–0.487)	0.428 (0.27 to 0.515)
Y	11.399 (8.984 to 13.146)	−0.157 (−0.242 to −0.087)	0.161 (0.154 to 0.168)	−0.972 (−1.525 to −0.541)	1 (1–1)	−2.905 (−3.419 to −2.208)	1.445 (1.369 to 1.585)	−2.009 (−2.405 to −1.461)	1 (1–1)	0.37 (0.29 to 0.457)
P	−8.966 (−11.058 to −5.903)	−0.106 (−0.157 to −0.032)	0.175 (0.16 to 0.187)	−0.607 (−0.873 to −0.189)	1 (1–1)	2.556 (1.662 to 3.16)	1.566 (1.464 to 1.688)	1.627 (1.084 to 2.001)	1 (1–1)	0.101 (0.049 to 0.155)
I	10.355 (8.192 to 12.409)	−0.267 (−0.328 to −0.213)	0.208 (0.193 to 0.227)	−1.283 (−1.581 to −1.032)	1 (1–1)	−2.604 (−3.165 to −1.979)	1.877 (1.73 to 2.057)	−1.393 (−1.72 to −1.028)	1 (1–1)	0.319 (0.223 to 0.417)
S	11.379 (9.526 to 14.311)	−0.021 (−0.077 to 0.022)	0.152 (0.14 to 0.163)	−0.138 (−0.503 to 0.144)	1 (1–1)	−3.004 (−3.817 to −2.501)	1.37 (1.285 to 1.48)	−2.203 (−2.704 to −1.838)	1 (0.653–1)	0.283 (0.232 to 0.365)
T	11.592 (8.601 to 17.096)	0.129 (0.064 to 0.218)	0.238 (0.211 to 0.273)	0.54 (0.294 to 0.829)	1 (1–1)	−3.328 (−4.89 to −2.449)	2.141 (1.902 to 2.45)	−1.552 (−2.068 to −1.242)	1 (1–1)	0.097 (0.068 to 0.146)
Q	−13.938 (−15.9 to −10.231)	0.226 (0.164 to 0.319)	0.139 (0.133 to 0.146)	1.625 (1.153 to 2.352)	1 (1–1)	3.562 (2.464 to 4.132)	1.245 (1.188 to 1.374)	2.872 (1.842 to 3.332)	0.43 (0.131–1)	0.571 (0.465 to 0.633)
G	−4.151 (−13.291 to −0.039)	0.212 (0.139 to 0.315)	0.249 (0.213 to 0.284)	0.858 (0.629 to 1.121)	1 (1–1)	1.029 (−0.08 to 3.519)	2.232 (2.051 to 2.496)	0.463 (−0.037 to 1.546)	1 (1–1)	0.105 (0.04 to 0.26)
E	−14.731 (−20.071 to −10.861)	−0.042 (−0.103 to 0.026)	0.219 (0.182 to 0.243)	−0.2 (−0.498 to 0.117)	1 (1–1)	4.026 (2.992 to 5.482)	1.947 (1.754 to 2.108)	2.07 (1.481 to 3.07)	1 (0.26–1)	0.215 (0.117 to 0.376)
D	−11.406 (−14.323 to −7.232)	−0.187 (−0.29 to −0.11)	0.178 (0.164 to −0.19)	−1.047 (−1.66 to 0.623)	1 (1–1)	3.231 (2.136 to 4.058)	1.597 (1.528 to 1.699)	2.024 (1.299 to 2.533)	1 (0.985–1)	0.131 (0.061 to 0.21)
H	7.231 (0.385 to 12.453)	−0.101 (−0.205 to −0.029)	0.245 (0.195 to 0.276)	−0.41 (−0.957 to −0.119)	1 (1–1)	−1.866 (−3.297 to 0.097)	2.191 (1.945 to 2.389)	−0.848 (−1.512 to 0.046)	1 (1–1)	0.095 (0.043 to 0.179)
K	−2.684 (−7.246 to 0.217)	0.111 (0.019 to 0.188)	0.216 (0.202 to 0.231)	0.511 (0.091 to 0.851)	1 (1–1)	0.576 (−0.238 to 1.898)	1.94 (1.827 to 2.102)	0.299 (−0.125 to 0.917)	1 (1–1)	0.042 (0.017 to 0.099)
W	11.713 (8.018 to 15.264)	−0.007 (−0.098 to 0.095)	0.21 (0.192 to 0.23)	−0.034 (−0.473 to 0.451)	1 (1–1)	−3.199 (−4.235 to −2.205)	1.895 (1.769 to 2.061)	−1.695 (−2.152 to −1.145)	1 (1–1)	0.19 (0.069 to 0.25)
C	7.395 (5.315 to 10.982)	−0.188 (−0.232 to −0.143)	0.162 (0.147 to 0.176)	−1.162 (−1.427 to −0.857)	1 (1–1)	−1.877 (−2.835 to −1.273)	1.459 (1.371 to 1.568)	−1.286 (−1.887 to −0.883)	1 (1–1)	0.282 (0.213 to 0.372)
N	−1.339 (−4.335 to 2.432)	−0.039 (−0.123 to 0)	0.163 (0.135 to 0.178)	−0.242 (−0.9 to 0.002)	1 (1–1)	0.508 (−0.495 to 1.392)	1.451 (1.336 to 1.536)	0.35 (−0.351 to 1.004)	1 (1–1)	0.007 (0 to 0.04)

*Note:* Estimates obtained from PGLS. Values in parentheses correspond to the interval of each parameter considering the topology of the trees (see Section 2).

* *p*‐values are adjusted to multiple comparison following the Benjamini–Hochberg method.

## 4 Discussion

Lepidopterans are cornerstone species for biodiversity conservation and ecosystem services (Noriega et al. 2018). Their thermal limit provides insights into their strategies to cope with fluctuating climates (e.g., Kingsolver and Watt 1983; Davies et al. 2006; Ashe‐Jepson et al. 2023). Likewise, their diet breadth can predict their ability to respond to varying environments and ascertain their aptitude to adapt and specialise to new hosts (e.g., Descombes et al. 2016; Hausharter et al. 2023; Seifert and Fiedler 2024). We presented the first comprehensive test for the hypothesis that amino acid frequencies in the proteome—the proteome composition—are associated with diet and temperature across Lepidopterans. This hypothesis is derived from previous literature in prokaryotes which showed that the amino acid frequencies in the proteome are dependent upon the optimal growth temperature of a species, raising the intriguing possibility that the proteome contains information about the thermal ecology of the organism (Dufton 1997; Chen and Nielsen 2022; Seligmann 2003; Singer and Hickey 2003; Tekaia and Yeramian 2006; Swire 2007). Our most recent comprehensive study tested this hypothesis across three domains of life (archaea, bacteria, eukaryotes) and found supporting evidence that the frequency of thermolabile amino acids such as cysteine in the proteome is negatively associated with optimal growth temperature (Morimoto and Pietras 2024). With the growing impacts of climate change, uncovering new relationships between species' biology and ecology—such as the link between proteome, temperature, and diet studied here—is paramount to help understand species' potential to tolerate increasing temperatures (see also Lancaster 2016). Our data revealed no evidence that amino acid frequencies in the proteome were associated with maximum temperature or diet breadth of the 35 Lepidoptera species. These results suggest that complex animal proteomes are likely shaped by energetic and biophysical constraints rather than by ecological factors portending to the temperature and diet niches which species can explore. We know that Lepidopterans respond to thermal conditions through plastic expression of heat‐shock proteins that ultimately leads to evolutionary adaptations at the genome (and consequently, proteome) level (Mutamiswa et al. 2023). Likewise, as they colonise new environments in response to thermal conditions, Lepidopterans also need to adapt to new host plants, resulting in additional pressures that also shape the genome and proteome. Our findings suggest that those changes are not linked to substantial changes in proteome composition, and that neither temperature nor diet breadth correlated with the frequency of any amino acid in the proteome.

Amino acid frequencies in the proteome contain information about dietary needs underpinning life‐history traits, but our data show that this does not necessarily apply to broader ecological traits. For instance, Piper et al. (2017) showed that *D. melanogaster* and *M. musculus* fed diets with amino acids that were proportional to the amino acid frequencies of the proteome (“exome‐matched” diets) optimise growth and reproduction without associated costs to lifespan. Gómez Ortega et al. (2023) corroborated this in *D. melanogaster* and showed that diets that matched amino acid proteome composition were better at improving fecundity from both sexes compared with diets with amino acids with transcriptome‐weighted proteome compositions. This suggests that differential gene expression does not influence the broader information on amino acid needs of the organism and that the proteome inherently contains information about organismal nutritional needs above and beyond gene expression levels. Together, these findings show that proteome composition informs dietary needs for life‐history traits. Given this, we hypothesised that the proteome could also contain information about how organisms interact with their environment, under the assumption that the relationships between proteome composition, diet, and optimal growth temperature extend to other ecological traits. Here, we tested these relationships using analogous ecological traits to those found to correlate with proteome composition, namely maximum temperature (analogous to growth temperature) and dietary niche breadth (analogous to dietary needs). Yet, our results found no evidence that proteome composition was associated with either of these ecological traits. Thus, it appears that proteome compositions are linked to intrinsic (physiological) organismal needs (optimal growth temperature and diet to realise life histories) but not with extrinsic (ecological) organismal responses (maximum temperature, dietary breadth). Whether this is a broader pattern across all multicellular organisms and across all ecological traits remains to be ascertained. Nonetheless, our findings show for the first time that, despite being linked to optimal growth temperature and dietary needs, proteome composition is not a good indicator of species thermal limits or dietary niches.

While innovative, our work has limitations and should be interpreted with caution. First, it is possible that the lack of statistical significance reported here stems from our relatively small (35 species) sample size. It is possible to increase power by better integrating databases. For example, if we prioritise data collection of ecological traits of species for which there is genome sequenced and annotated, we can have a complete picture of how ecology and genomics interact. This would benefit the field of comparative ecological genomics by enabling integrative approaches such as the one conducted here. A second caveat worth highlighting is the use of the maximum temperature in which the species are found—effectively their *realised* thermal limit—as an ecological trait for correlations with proteome composition. Even though there is a strong phylogenetic signal in species' upper thermal limit in insects (see e.g., Hoffmann et al. 2013), our occurrence data could reflect a constrained distribution pattern where individuals occur below their limits. Moreover, microclimatic factors could also add uncertainty to our estimates of species' true realised thermal limits. Unfortunately, thermal physiological limits have not been estimated experimentally for many Lepidoptera species—including those used in this study (Diamond et al. 2024). Moreover, we lack a comprehensive database of microclimatic temperatures for most insects even though microclimate plays a key role in insect persistence in climate change (Kerr et al. 2025). With improved remote sensing technologies (Zellweger et al. 2019), we expect to have microclimatic data which will allow us to test how micro‐ and macro‐climate correlate or not with proteome composition. Despite these limitations, our methodology is robust and makes use of the most comprehensive integrated database available to test our hypotheses at the time. We hope our approach will inspire future integrative approaches such as this—and the efforts to expand the underlying databases which make them possible.

## 5 Conclusion

Proteomes are a fundamental feature of biological systems and inform about the global amino acid requirements of a genome. Our work on Lepidoptera suggests that proteomes are largely independent of observable ecological traits related to thermal limits and diet breadth. This allows for the possibility that proteomes are primarily shaped by evolutionary constraints and do not contain signatures of ecological conditions in which species exist. This knowledge expands our understanding of evolutionary versus ecological constraints in proteome composition and partly contradicts evidence from prokaryotes and unicellular eukaryotes which showed that some ecological traits influence proteome composition. Multicellular organisms are complex and might rely on more stable proteome compositions to achieve the multitude of physiological control and homeostasis across environments. Future studies in other taxonomic groups will provide valuable tests of this hypothesis.

## Author Contributions

**Zuzanna Pietras:** conceptualization (supporting), investigation (supporting), project administration (supporting), writing – original draft (supporting), writing – review and editing (supporting). **Fernanda S. Caron:** conceptualization (equal), data curation (equal), formal analysis (equal), investigation (equal), writing – original draft (equal), writing – review and editing (equal). **Arkan Eddine‐Lomas:** investigation (equal), methodology (equal), software (lead), writing – original draft (supporting), writing – review and editing (supporting). **Rebecca von Hellfeld:** writing – original draft (supporting), writing – review and editing (supporting). **Juliano Morimoto:** conceptualization (lead), data curation (equal), formal analysis (supporting), investigation (supporting), methodology (lead), project administration (lead), supervision (lead), visualization (supporting), writing – original draft (equal), writing – review and editing (equal).

## Funding

J.M. was supported by the Biotechnology and Biological Sciences Research Council (BBSRC: BB/V015249/1). F.S.C. was funded through a graduate scholarship from the Fundação Coordenação de Aperfeiçoamento de Pessoal de Nível Superior (CAPES, Grant 88887.923452/2023‐00).

## Conflicts of Interest

The authors declare no conflicts of interest.

## Supporting information


**Table S1:** Accession numbers of the COI sequences used in the phylogenetic reconstruction.
**Table S2:** Calibration scheme used in the phylogenetic reconstruction based on Kawahara et al. (2023).

**Data S1:** ece372608‐sup‐0002‐Supinfo02.csv.

## Data Availability

GBIF occurrence data can be downloaded here: https://doi.org/10.15468/dl.q74xtz. Code for orthologous analysis can be found in https://github.com/Arkaned/lep_exome_analysis.git.
